# Setting Housing Standards to Improve Global Health

**DOI:** 10.3390/ijerph14121542

**Published:** 2017-12-09

**Authors:** Philippa Howden-Chapman, Nathalie Roebbel, Elinor Chisholm

**Affiliations:** 1 He Kainga Oranga, Housing and Health Programme, University of Otago, Wellington 6242, New Zealand; elinor.chisholm@otago.ac.nz; 2 WHO, Department of Public Health, Environmental and Social Determinants of Health, 1202 Geneva, Switzerland; roebbeln@who.int

**Keywords:** housing, health, methodology, international guidelines, WHO

## Abstract

Developing World Health Organization international guidelines is a highly formal process. Yet the resulting guidelines, which Member States are encouraged, but not required to adopt, are a powerful way of developing rigorous policy and fostering implementation. Using the example of the housing and health guidelines, which are currently being finalised, this paper outlines the process for developing WHO guidelines. This includes: forming a Guidelines Review Group that represents all regions of the world, and ensures gender balance and technical expertise; identifying key health outcomes of interest; commissioning systematic reviews of the evidence; assessing the evidence; and formulating recommendations. The strength of each recommendation is assessed based on the quality of the evidence, along with consideration of issues such as equity, acceptability, and feasibility of the implementation of the recommendation. The proposed housing guidelines will address: cold and hot indoor temperatures, home injuries, household crowding, accessibility and access to active travel infrastructure.

## 1. Introduction

One of the core functions of the World Health Organization (WHO) is its normative work: developing guidelines to assist policymakers, providers and recipients of health care, and other stakeholders to make informed decisions. WHO guidelines are based on formal systematic reviews of evidence, and may relate to clinical interventions, public health activities, or government policies. Guidelines are created through a transparent process to avoid conflict of interest, thereby assisting policymakers and other community actors to ensure their actions support health.

Since 2010, WHO has been engaged in a process to develop Housing and Health Guidelines. These will be the first WHO guidelines to focus on a sector as opposed to a specific health risk, intervention, activity or policy and thus detailing the methodological approach is fundamentally important. There were a number of rationales for producing guidelines on housing and health. The guidelines respond to the prevalence of poor housing conditions across both low-income and high-income countries [1,2,3,4,5,6], to the high burden of disease associated with issues related to housing [7,8,9] and to the increasing body of evidence that improving housing conditions improves health [10,11]. The guidelines bring together existing WHO guidelines on housing issues together with new evidence-based recommendations on healthy housing conditions and interventions. Accessible guidance on healthy housing conditions will enable health considerations to inform housing, energy, community development, and urban development policies. In sum, the guidelines are intended to support and promote international action on housing and health.

The WHO guideline development process is the same, regardless of whether WHO guidelines address specific public health issues, clinical interventions and policies, or a sector-wide approach [12]. Simply put, WHO decides to produce guidelines and assembles a Guideline Development Group (GDG). This group meets to identify key topics of interest and formulates key questions that form the basis of systematic reviews. The GDG assesses the evidence accumulated by the systematic review teams and formulates key recommendations. The guidelines then undergo an external and internal peer review process and then finally need to be approved by the WHO’s Guideline Review Committee. Throughout this process the GDG are supported by a steering group of experts from different WHO departments.

Currently the Housing and Health Guidelines have been submitted and are under review. In this article we outline the methodological issues raised by the development of these Guidelines. We provide an overview of the process for formulating the Housing and Health Guidelines, outline the key areas the guidelines focus on, and consider their implementation and their international relevance.

## 2. Developing the Guidelines

The process for developing new Housing and Health Guidelines was guided by the WHO secretariat. WHO assembled a Guideline Development Group (GDG). The GDG consists of people with expertise in housing and health, provides for regional diversity and gender balance, and includes people with expertise in evidence-based guideline development. The GDG first met in Washington DC in April 2013 to identify priority topics for the guidelines. This made clear the need for eight distinct systematic reviews of the effects of the relevant interventions or exposures: crowding, low indoor temperatures, insulation, energy affordability, high indoor temperatures, injury hazards, housing accessibility, and proximity of housing to active travel infrastructure. Members of the GDG prepared scoping papers on these as well as a number of additional exposures and interventions related to housing and health. Although the GDG acknowledged the importance of other housing issues to health—such as housing affordability, secure occupancy, and the accessibility of housing to green space, public transport and community resources—these were considered of lesser priority, with a less developed evidence base, than the eight selected interventions or exposures. The GDG suggested these be considered in future guidelines.

The questions to be addressed by each systematic review were agreed by the WHO Secretariat and the GDG. These were converted into a PICO or PECO format. This acronym refers to Population, Intervention (or Exposure), Comparator and Outcome—four elements that should be considered in any question governing a systematic search of the evidence [12]. The GDG agreed on key health outcomes of interest for the systematic reviews, as well as the scope and eligibility criteria. The systematic review teams retrieved potentially relevant evidence, and assessed the quality of the evidence, in consultation with the WHO Secretariat and an expert methodologist. The quality assessment of the evidence gathered for the effects of the intervention or exposure on each of the prioritised health outcomes used the Grading of Recommendations Assessment, Development and Evaluation (GRADE) approach [12]. This enables the categorisation of the quality of evidence for each outcome, based on the study design, the risk of bias in the included studies, inconsistency of results, indirectness, imprecision, and a number of other relevant factors.

A number of studies were not eligible for systematic review because they reported the results of multifactorial interventions, which aim to address a number of housing risks at the same time. This is a practical, efficient and cost-effective approach often taken by researchers as well as public and private organizations that rehouse people or rehabilitate housing. This type of intervention, which for example may include reducing crowding, installing safety measures, and improving ventilation and heating, and educating occupants, has been shown to reduce the likelihood of hospitalisation (for examples, see [13,14]). However, from the methodological point of view, this approach makes it difficult to ascribe a particular health effect to a particular part of the intervention. The GDG discussed at length the difficulty of teasing apart the individual contributions in multi-pronged public health interventions, while recognising that discounting such studies may under-count the influence of multiple, unmeasured variables, which could have a causative influence. Due to these challenges of systematically reviewing multifactorial interventions (see discussion in [15]) and following the advice of the Guideline Review Committee, it was decided to exclude this type of study from the systematic reviews. However, examples of multi-pronged interventions are given in the implementation chapter. If Guideline protocols are revised to accommodate a broader public health approach, future guideline iterations may also be able to include this type of evidence.

The GDG noted that a number of issues relating to housing and health were already covered by existing WHO guidelines. These address the quality of the water accessible to people in their homes or nearby; the quality of the air in the home, including relationships to indoor fuel and other pollutants, dampness, mould, tobacco smoke, and radon; neighbourhood noise; and the presence of toxins in the home such as asbestos and lead. The GDG elected to provide summaries of existing guidelines as well as to develop new guidelines, so that all WHO guidelines relating to health will be available in the same document. The guidelines also provide extensive discussion of the importance of social and economic factors in determining the type of housing available to people, and whether this housing harms their health.

At the GDG’s second meeting, in Morges (Switzerland) in July 2015, the GDG drew on the systematic reviews, and the information on the quality of the evidence, to initiate their discussion of the recommendations for each of the topics. Each recommendation was defined as either strong or conditional, based on the evidence from the relevant systematic review, along with information about balance between benefits and harms, values and preferences, equity, acceptability, feasibility and resource implications for the implementation of the recommendation. Strong recommendations are those that the GDG think should be implemented in most circumstances. The GRADE Evidence to Decision (EtD) framework for public health was used to formulate each recommendation [16].

When reviewing the systematic review on energy affordability, it was decided that there was insufficient direct evidence on the prioritised health outcomes to formulate a recommendation. For example, the review results provided weak evidence that residents with access to non-polluting indoor household energy are associated with better health outcomes than residents not able to afford non-polluting indoor energy. Most studies looking at the burden of disease associated with energy access and energy efficiency of housing look into the impacts of risks and conditions that are triggered by energy access problems, such as temperature or insufficient ventilation. Many of these studies focus on indoor cold, indoor air quality and ventilation, fuel combustion, dampness and mould, but not the effect of energy affordability per se. While the energy affordability discussions provided an important context for the GDG recommendations about high and low indoor temperatures, and are included in the text of the guidelines, additional research would be needed to develop robust recommendations on energy affordability.

The proposed housing and health guidelines have been reviewed by external reviewers, who were drawn from subject experts, implementing agencies and partners working on various aspects of policy to improve health outcomes related to housing. The reviewers included representatives of the health sector in a number of member states, as well as representatives of key groups such as tenants and architects. Reviewers’ comments were used to revise the guidelines. Subsequent to the peer review process, the guidelines are being considered by the Guideline Review Committee and if approved will be finalised. In what follows, we outline the importance of each of the topics addressed by the new guidelines, and the systematic review process.

## 3. Results

### 3.1. Inadequate Living Space (Crowding)

Crowding exists where the number of occupants in a home exceeds the capacity of the dwelling space available, whether measured as rooms, bedrooms or floor area [17,18]; whether crowding is occurring can depends on the relationship, gender and age of occupants [19,20,21]. Worldwide, crowding is often a marker of poverty and social deprivation [22,23]; it is a defining characteristic of slum housing [24]. Crowding is associated with exposure to infectious disease. Approximately 10% of hospital admissions per year in New Zealand are attributable to household crowding [9]. In Europe, household crowding is associated with more than 3500 deaths from tuberculosis per year [7]. In addition, crowding increases exposure to other risk factors, including home injury, social tensions and the likelihood of exposure to second-hand smoke [25,26].

The systematic review was based on the following question: In the general population exposed to household crowding, what is the exposure-response relationship between exposure to crowding and the proportion of persons with poorer health compared to the population not exposed to household crowding? The systematic review resulted in a large number of studies concerning crowding and tuberculosis, gastroenteritis and diarrheal diseases and other infectious disease, and mental health [27]. Overall, there was sufficient evidence to make a guideline about crowding.

### 3.2. Low Indoor Temperatures

Cold indoor temperatures are a consequence of outdoor temperature, structural deficiencies, including a lack of insulation, and lack of heating. Cold homes contribute to excess winter mortality and morbidity due to respiratory and cardiovascular illnesses. It is estimated that excess winter deaths (EWD) due to cold housing was 38,200 per year (12.8/100,000) in 11 selected European countries [7]. Winter mortality is greater in countries with milder climates than in those with more severe winter conditions, which suggests that countries with mild winters often have homes characterized by poor thermal efficiency that are harder to heat than well insulated houses in more extreme climates [28] Socio-economic factors and energy affordability play an important role in determining whether a dwelling is sufficiently warm [29].

In order to establish clear guidance on minimising the health risk associated with cold indoor temperatures, two systematic reviews of the evidence were commissioned. One systematic review looked at whether people living in housing where indoor temperatures are below 18 °C have worse health outcomes than those living in housing with indoor temperatures above 18 °C. The second systematic review focussed on whether people living in housing with insulation have better health outcomes than those living in housing without insulation. The systematic reviews resulted in studies considering the effects of indoor cold on respiratory and cardiovascular morbidity and mortality, and the effects of weatherisation and insulation on health outcomes [30,31]. There was sufficient evidence to make guideline recommendations about cold indoor temperatures and insulation.

### 3.3. High Indoor Temperatures

Public health interest in the effects of heat on health has grown in part because of the increasing frequency and duration of heat waves, which are considered to be an effect of climate change [32]. It is estimated that an excess of 70,000 people in 16 countries across Europe died in August 2003 due to a major heat wave [33]. People who live in temperate climates are more likely to be affected by high temperatures; the temperature threshold where heat-related deaths begin to increase during a heat wave is lower in cities with cooler climates [34,35]. Complete acclimatisation to an unfamiliar thermal environment may take several years [36,37].

In order to establish clear guidance on minimising the health risk associated with high indoor temperatures, a systematic review of the evidence was commissioned to identify whether people living in housing where indoor temperatures are above 24 °C have worse health outcomes than those living in housing with indoor temperatures below 24 °C [38,39]. Due to the small number of studies that resulted from the systematic review, additional analysis was conducted to provide indirect evidence of the relationship between high indoor temperatures and health outcomes. This drew on the association between high outdoor temperatures with high indoor temperatures, and the evidence that high outdoor temperatures harm health [40]. There was sufficient evidence to make a guideline recommendation about high indoor temperatures.

### 3.4. Injury Hazards in the Home

Injuries in the home present an important health burden worldwide [41]. Globally around a third of injuries occur in the home [42]. In Europe alone, almost 110,000 people die each year as a result of an injury sustained at home or at leisure, and an estimated 32 million are hospitalized [43]. Falls account for the largest proportion of the injuries in the home that require medical attention [44]. In Europe in 2010, around ten deaths (0.007 per 100,000) and 3310 DALYs (2.0 per 100,000) were attributable to a lack of window guards [7]. In India in 2005, the rate of deaths from falls in urban areas was 15.6 per 100,000; falling from stairs and ladders accounted for 8% of these [45].

In order to establish clear guidance on minimising the health risk associated with hazards in the home, a systematic review of the evidence was commissioned. This looked at whether residents in housing with fewer hazards have fewer injuries than residents living in housing with more hazards [46]. There was sufficient evidence to make a guideline recommendation about safety interventions in the home. 

### 3.5. Accessibility of Housing for People with Functional Impairments

At least a billion people, or 15% of the world’s population, have some form of disability [47]. The disabled population is increasing as the world population ages, because older people have a higher risk of disability [48]. Disability is an umbrella term describing physical or psychological impairments, activity limitations or participation restrictions [49]. Modifying features of the social and physical environment can reduce the severity and incidence of disability in a population. In the case of housing, for example, modifications such as installing ramps, widening doorways and lowering benchtops, can help enable wheelchair users to live independently and participate fully in all aspects of life [49,50]. Ensuring accessibility in the home environment, as the place where people spend most of their time [51], is vital both to fulfilling the human rights of disabled people and to public health [52].

In order to establish clear guidance on maximising the health gains associated with accessible housing, a systematic review of the evidence was commissioned. This looked at whether residents with functional or cognitive impairments living in accessible home environments have better health and social outcomes than residents with functional or cognitive impairments living in conventional or unmodified home environments [53]. There was sufficient evidence to make a guideline recommendation concerning accessible housing.

### 3.6. Proximity of Housing to Walking and Cycling Infrastructure

The problems of declining physical activity and the negative impact on health outcomes has been widely investigated and reported in the literature [54]. Physical inactivity is an important risk factor for non-communicable diseases, causing 6% of all deaths globally [55]. Travelling by active means has positive effects on the health of active travellers, especially if travel occurs over longer periods and over longer distances [56,57]. Active travel is associated with reducing the risk of cardiovascular disease [58], mortality [59,60] and obesity [61]. Individual factors influence whether people walk or cycle: age, health, weather, the distance of the destination, the terrain (including steepness of inclines), the need to transport passengers or heavy loads, cultural norms, access to private motorised transportation, and the availability, efficiency, and affordability of public transport options. In addition, urban planning and transport policies can have an important impact on physical activity [62,63,64,65,66].

Some aspects of the built environment can make walking or cycling more enjoyable, safer and practical: neighbourhood safety and appeal, road and pavement quality, secure and convenient cycle parks, street lighting, traffic volume on roads, and the availability of cycle lanes and walking trails [67,68,69,70,71]. In order to better understand the relationship between where people live and how they travel, a systematic review was commissioned. This looked at whether residents with closer access to walking or cycling infrastructure have better health behaviours and health outcomes than those who live further away from, or who do not have easy access to walking or cycling infrastructure [72]. There was sufficient evidence to make a guideline recommendation about the proximity of housing to cycle and walking infrastructure.

These systematic reviews assessed the state of the evidence around key issues related to housing and health, and pointed towards priorities for future research. The recommendations that result will give clear guidance to policymakers on how housing can support health. In the next section, we anticipate the acceptance of the guidelines and consider factors affecting the implementation of the guidelines.

### 3.7. Implementing the Guidelines

The proposed Housing and Health Guidelines address diverse areas: indoor temperature; the presence of injury hazards; whether the home is accessible and provides adequate space for its occupants; and where it is located in relation to walking and cycling infrastructure. Implementing the guidelines therefore is likely to involve a diverse array of interventions and involve a number of actors, including owners and occupants, community organizations, builders, developers and planners, and government. Intra-governmental coordination is imperative. Both local and central governments may build, rent out or help finance housing. Central government action clearly affects housing supply and demand, and may be responsible for regulation and subsidies intended to encourage healthy housing. Local government may have responsibility for land supply, building codes and consents, infrastructure such as connection to utilities, roads, cycle ways and public transport, as well as enforcement of regulations related to sanitation and standards in existing dwellings. The health sector can play an important role in assessing and communicating health risks and benefits associated with housing, and can provide tools to help policymakers and the private sector integrate health concerns into planning and construction. The health sector should therefore be involved in housing policy at all levels.

The extent to which Housing and Health Guidelines are applicable to different contexts was discussed throughout the guideline development process. At Habitat III in Quito in October 2016, the United Nations Human Settlement Programme (UN-HABITAT) and WHO co-hosted a meeting focussed on the guidelines in slum and informal housing. The guidelines are intended to be internationally applicable, and to state the necessary conditions required to maintain health and prevent housing-related health conditions. They provide recommendations relevant to everyone, including people living in slums and informal settlements. WHO guidelines—new and old—address some of the most urgent risk factors relevant to slums, such as indoor air quality, water quality, and crowding. However, implementing the HHGL is likely to be much more challenging in informal housing. Ameliorating urgent health risks—such as unsafe electric connections, or getting people out of severely polluted or dangerous areas—is necessary alongside (or prior to) working on bringing housing up to the guideline recommendations. The amount of resources available will determine what kinds of action can be taken. In some cases, the guideline recommendations will not be fully met, but they will work as a target. For example, reducing crowding or reducing the number of hazards in a home will be steps towards reducing the risks to health associated with housing. The guidelines can also be used to support the prioritisation of slum upgrading interventions. Consideration of interventions to contribute to improving housing in slums can be based on the strength of the guideline recommendation alongside consideration of resource constraints.

## 4. Conclusions

Creating WHO guidelines is a challenging process, designed to ensure recommendations are robust. This paper has set out the process for developing Housing and Health Guidelines, including the identification of key topics, the commissioning of systematic reviews, and the grading of evidence and recommendations. The guidelines have undergone public peer review before being considered by the WHO’s Guideline Review Committee. When this process is successfully completed, the recommendations and best practice statements contained in these guidelines will be disseminated with the cooperation of a broad network of international partners, including: WHO country and regional offices; ministries of health; ministries of building and construction; WHO collaborating centres; other United Nations agencies, particularly the United Nations Human Settlement Programme (UN-HABITAT); and non-governmental organizations. They will also be available on the WHO website. In addition, an executive summary aimed at both health professionals and professionals of the construction and building sector and a wide range of policy-makers and programme managers will be developed and disseminated through WHO country offices and their respective partners.

Based on WHO’s approval, web-based guidance and tools that build on the evidence reviews used to inform these guidelines will be prepared. WHO will work with member states to support the implementation process through its regional and country offices. This includes healthy housing checklists, model interventions, legislation and regulation, and capacity building and information tools. Guidance will be developed engaging with different sectors and on how to prioritize actions on housing and health in slums and other informal settlements. WHO will work closely with some of the countries most affected by substandard housing conditions to learn from initial stages of implementation, and will use this experience to revise the guidance and tools. Monitoring and evaluation, which is vital to understanding the extent of housing problems, and which will provide an indication of the impact of the guidelines. One way for monitoring the impact of the guidelines is by applying the Environmental Burden of Disease (EBD) approach for healthy homes [7].

The Housing and Health Guidelines will be responsive to new evidence and new information needs. The recommendations can be updated as more evidence becomes available. The updated edition can also address some key housing risk factors not covered by this edition. This could include access to green and public spaces, vector borne diseases and pests, security of occupancy or tenure, residential mobility, accessibility to transport options, and accessibility to additional elements in the built environment that promote active travel. In addition, WHO could provide concrete recommendations on multi-factorial interventions.

Through the Housing and Health Guidelines, WHO can continue to support member states in their efforts to promote healthy housing and to reduce the burden of disease associated with housing. Healthy housing conditions are essential for achieving the Sustainable Development Goal 11 “to make cities safe, inclusive, resilient, and sustainable” [73]. The guideline development process set out in this paper is fundamental to ensuring recommendations are robust. Once the guidelines are approved, supporting communities and governments to implement the guidelines, and supporting monitoring and evaluation of housing conditions, are crucial next steps for WHO.
